# Global Evidence on Helmet Use and Misuse: A Public Health Perspective on Prevalence, Determinants and Barriers

**DOI:** 10.1002/hsr2.72078

**Published:** 2026-03-29

**Authors:** Ramin Rezapour, Reza Ghorbani, Naser Derakhshani, Leili Abedi, Mahdi Sayyadzadeh, Mohammad Saadati

**Affiliations:** ^1^ Student Research Committee Tabriz University of Medical Sciences Tabriz Iran; ^2^ Tabriz Health Services Management Research Center Tabriz University of Medical Sciences Tabriz Iran; ^3^ Road Traffic Injury Research Center Tabriz University of Medical Sciences Tabriz Iran; ^4^ Health Management and Economics Research Center Iran University of Medical Sciences Tehran Iran; ^5^ Faculty of Medicine Bam University of Medical Sciences Bam Iran; ^6^ Department of Environmental Health Engineering School of Health Tabriz University of Medical Sciences Tabriz Iran; ^7^ Department of Public Health Khoy University of Medical Sciences Khoy Iran

**Keywords:** barriers, determinants, helmet use, motorcyclists, systematic review

## Abstract

**Background:**

Helmet wearing is crucial to prevent injuries which is influenced by diverse variables. There are some factors which affect helmet use or non‐use resulting in fluctuations in helmet use rate, globally. This study aimed at estimating global helmet use rate and identifying related barriers and determinants.

**Method:**

A systematic review and meta‐analysis was performed till January 2024 through PubMed, Scopus, ScienceDirect, and Web of Knowledge databases using predefined keywords. Related journals and references of the selected articles were reviewed. Two authors independently screened the retrieved citations, assessed methodological quality, and extracted data using an extraction form. Subgroup analysis and sensitivity analysis were performed to explore differences based on study characteristics, and random effect model was used for meta‐analysis through Stata 16.

**Result:**

Based on meta‐analysis results, including approximately one million people, overall helmet use rate (OHUR) in motorcyclists and passengers were estimated to be 0.54 [0.48–0.59 with 95 CI, I^2^ = 99.9] and 0.32 [0.15–0.49 with 95 CI, I^2^ = 99.9], respectively. Correct helmet use rate (CHUR) in motorcyclists was 0.43 [0.32–0.53 with 95 CI, I^2^ = 99.9] and 0.18 [0.12–0.24 with 95 CI, I^2^ = 9] in passengers. Identified determinants and barriers were classified in eight categories, including psychological and cognitive, socio‐demographic, travel characteristics, road characteristics, motorcycle, time/weather condition, law enforcement, and miscellaneous.

**Conclusion:**

Although approximately half of the motorcyclists and a third of the passengers have used helmet, the correct use was very low. Psychological and cognitive factors were the most important barriers to helmet use. Creating and maintaining a positive attitude in motorcyclists about safety effects of helmet through diverse methods such as peer‐groups education, social campaigns, and so forth is recommended. Moreover, developing compatible, comfortable, and affordable helmets should be followed to overarch barriers.

## 1 Background

Motorcyclists are vulnerable road users with high risk of crash and injury, especially in low‐middle income countries (LMICs) [1]. They often share the traffic space with other fast‐moving vehicles and are less visible. In addition, their lack of physical protection makes them more vulnerable to injury [2]. Injuries to the head and neck were raised as the main cause of death, severe injury, and disability among motorcyclists [3]. Head injuries also result in much higher medical costs than any other type of injuries. In other words, these injuries exert a high toll on a country's health care costs and its economy [1]. Literature revealed that wearing a motorcycle helmet can reduce death risk by almost 40% and severe injury risk by approximately 70% [1]. Effective enforcement of motorcycle helmet law can increase helmet‐wearing rate and thereby reduce injuries [4]. Helmet law should apply to all riders (including children) and specify a helmet‐quality standard, but only 49 countries (representing 1.2 billion people) have such comprehensive law on motorcycle helmet [5].

A review found that across ASEAN countries, a considerable proportion of motorcycle drivers did not use a helmet; this ranged from 11% to 20% in Indonesia, 35% to 66% in Cambodia, 25% to 97% in Laos, 24.2% to 67.2% in Malaysia, 44.2% to 56.3% in Thailand, and 10% to 70.1% in Vietnam. These rats were higher in passengers [6]. A study by Fong et al. (2015) showed that only 16.2% of adult motorcyclists use helmets. When asked about helmet use attitudes, the majority of adults indicated that they did not like how helmets feel or made them look [7]. In Tanzania, helmet use was reported to be 82.1% among drivers and 22.5% among passengers [8]. Helmet heaviness, warming up inside the helmet, the length of trip, negative attitude toward the safety impact of helmet, existence of the mandatory law, driving experience, age and sex, type of road, and type of motorcycle had been expressed as effective factors in helmet use in other studies [4, 9, 10, 11, 12, 13]. Given that preventing traffic crashes and reducing injuries is an international priority based on sustainable development goals (SDGs) [14], identifying individual and systematic factors affecting the safe behavior of users could provide useful information for adopting appropriate policies. Regarding, this study aimed at systematically estimating motorcycle helmet usage and its barriers and facilitators.

## 2 Methods

This is a systematic review and meta‐analysis study conducted till January 2024. We used Preferred Reporting Items for Systematic Reviews and Meta‐Analyses (PRISMA) guidance to design the study and outline the final report [15].

### 2.1 Literature Search

Primarily, keywords were obtained from MeSH, related literature, and experts' opinions, and were finalized through a pilot search. The search was conducted with time limitation from 1995 to January 2024.

PubMed, Scopus, ScienceDirect, and Web of Knowledge databases were searched using determined keywords (Helmet, Helmet use*, predictor, determinant, Barrier, affecting factor, and facilitator) for English literature (Appendix 1). Moreover, beside manual search of related journals, references of the included articles were also reviewed to increase the chance of articles finding.

### 2.2 Screening and Data Extraction

EndNote X9 software package was used to organize, and screen retrieved citations. First, duplicates were excluded, then articles were screened by reviewing their titles to exclude non‐relevant citations. Then abstracts were studied and some of the articles were excluded because of non‐reporting of the helmet using rate, barriers or facilitators. Remained citations were reviewed based on full text and articles were included based on inclusion criteria. The screening process was done independently by two of authors (L.A. and M.S.) and disagreements were solved by third one (N.D.). Extraction table was used to extract the data, including name of author/s, publication year, country, data collection methods, barrier/determinants of helmet use, and helmet use rate among motorcycle riders/passengers. The full data extraction sheet is available in Appendix 2.

### 2.3 Inclusion and Exclusion Criteria

#### 2.3.1 Inclusion Criteria

- Articles with publishing date after 2000.
- Articles reporting both the barriers or facilitators and helmet using.
- Articles reporting the helmet use rate among riders/passengers.
- Observational studies (to be included in meta‐analysis).

#### 2.3.2 Exclusion Criteria

- Studies that pointed to a specific population, such as children or the elderly.
- Conference abstracts and articles which had reported the effects of helmet.
- Studies that considered different calculation criteria for helmet use.
- Studies that categorized participants based on how long they wore a helmet (always, often, occasionally, rarely, or never).
- Interventional studies, conference papers, and short communication.
- Publishing date before 2000.
- Studies on all‐terrain vehicle motorcycles.

### 2.4 Critical Appraisal

Critical appraisal was done for 41 included articles in meta‐analysis. The studies reporting quality was independently assessed by two investigators (R.R. and R.G.) according to 22‐item STROBE checklist [16, 17]. This checklist contains 22 questions, but according to the cross‐sectional nature of the most included articles, 4 items related to case‐control and cohort studies were removed and eventually 18 remaining questions were evaluated. The checklist items score was 0, 1, and 2, according to matching the checklist question criteria with the contents of the articles. The minimum score for checklists was 0 and maximum 36. Studies were classified in good [the score in the range 25–36], medium [13, 14, 15, 16, 17, 18, 19, 20, 21, 22, 23, 24], and poor [0–12] quality studies.

Articles were assessed by two of the authors and they categorized studies into three groups of high, moderate, and low quality based on their overall score. Any disagreements between investigators were resolved by discussion and in consultation with the research team.

### 2.5 Data Analysis

#### 2.5.1 Qualitative Data

Content analysis was used to analyze the data, which is a method for identifying, analyzing, and reporting the themes within the text. It is also widely used in text data analysis.

#### 2.5.2 Quantitative Data

First, the data were entered in Excel sheet. Two variables of the overall use of helmets and the correct use of helmets in both groups of motorcyclists and passengers were estimated. Weighted average was estimated for studies that reported helmet use by city, season, or different areas of city. Moreover, sub‐group analyzes were performed based on data collection methods (self‐reported/observed).

Q and I^2^ were used to evaluate the heterogeneity of the studies' result. To interpret the I^2^, 25% was considered for low heterogeneity, 50% for medium, and 75% for high heterogeneity. Due to the high heterogeneity of the studies, random effect model was used for meta‐analysis. Forest plot diagram was used to report the results, where the size of each square represents the sample size and the lines on each side of the square represents 95% confidence interval for each study. Funnel plot diagram and Egger's regression test were used at a significance level of 0.1% to measure publication bias. Stata 16 was used to perform the meta‐analysis.

### 2.6 Patient and Public Involvement

It was not appropriate to involve patients or the public in the design, or conduct, or reporting, or dissemination plans of our research.

#### 2.6.1 Generative AI Use

We have used ChatGPT Graphical abstract designer to create our Graphical abstract along with the complete review by the authors.

## 3 Results

**FIGURE 1:**
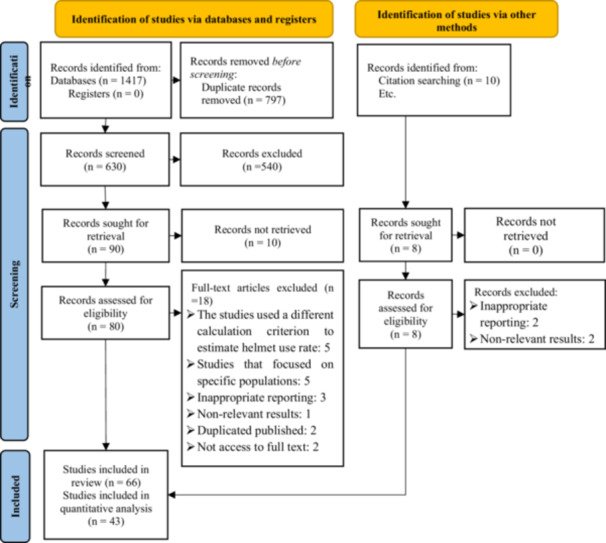
Searching and screening process.

A total of 1427 studies were retrieved in literature search process. After removing duplicates (*n* = 797), remained were screened based on title and abstract. About 88 articles were reviewed for eligibility and finally 66 were included in the qualitative synthesis and 43 articles of that were included in quantitative analysis. The study screening and selection process reported in PRISMA flow‐diagram (Figure 1).

Most of the included studies (*n* = 43 from 66) were published between 2010 and 2020. The studies were conducted in 19 different high, middle, and low‐income countries. Included studies (*n* = 66) characteristics are presented in Appendix 2. Meta‐analysis was run based on data from 1,031,268 people, 98.23% (1,013,091 people) of whom were motorcyclists.

### 3.1 Overall Helmet Use Rate (OHUR)

**FIGURE 2:**
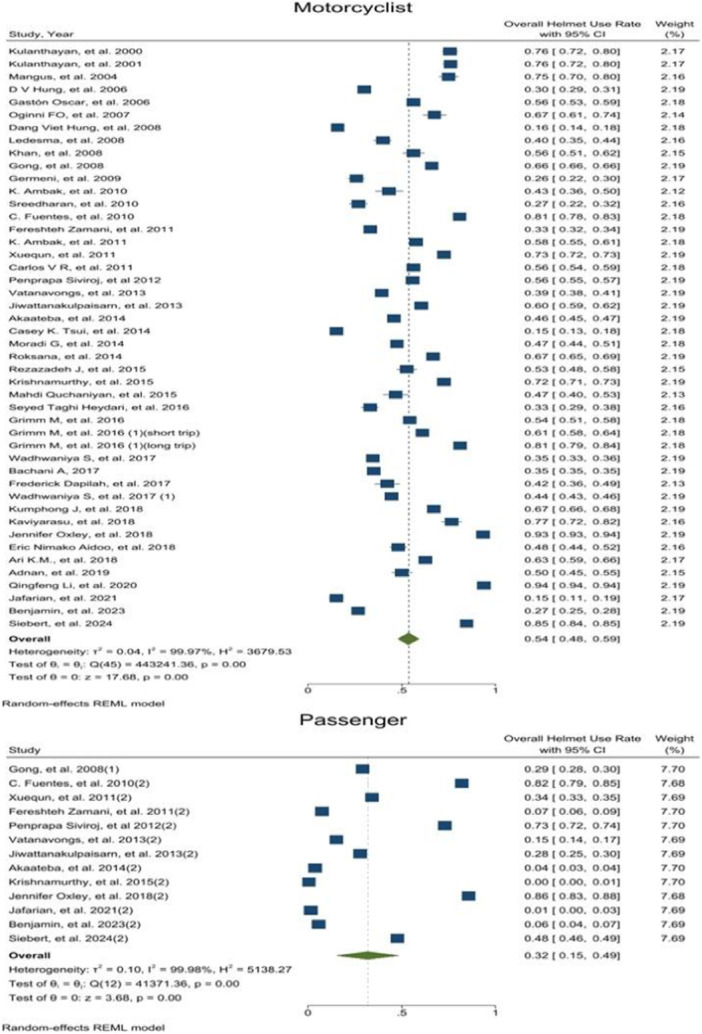
Overall helmet use rate based on motorcyclist and passenger.

OHUR in motorcyclists and passengers was reported by 46 and 13 studies, respectively. OHUR in motorcyclist was estimated to be 0.54 [0.48–0.59 with 95 CI, I^2^ = 99.9] and in passengers it was 0.32 [0.15–0.49 with 95 CI, I^2^ = 99.9]. Results revealed a high level of heterogeneity among the study's results. Moreover, publication bias test results showed that the probability of publication bias was very low (Publication Bias‐ Egger test, *p*‐value = 0.34, *p*‐value = 0.21) (Figure 2) (Appendix 3). Also, the helmet use rate among motorcyclists was significantly higher than that of passengers (*p*‐value < 0.001).

### 3.2 Correct Helmet Use Rate (CHUR)

**FIGURE 3:**
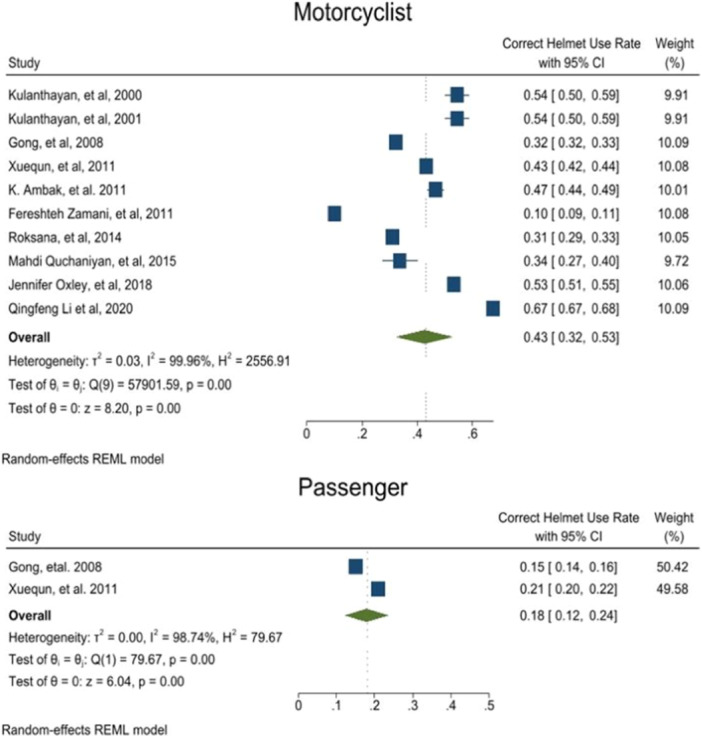
Correct helmet use rate based on motorcyclist and passenger.

CHUR in motorcyclists based on 10 articles was estimated to be 0.43 [0.32–0.53 with 95 CI, I^2^ = 99.9] and for passengers was 0.18 [0.12–0.24 with 95 CI, I^2^ = 9] based on 2 studies. High level of heterogeneity was observed. Moreover, a moderate probability of publication bias was revealed (Publication Bias‐ Egger test, *p*‐value = 0.17) (Figure 3) (Appendix 3). Also, the CHUR among motorcyclists was significantly higher than that of passengers (*p*‐value < 0.001).

### 3.3 Helmet Use Rate Based on Data Collection Methods Subgroups

**FIGURE 4:**
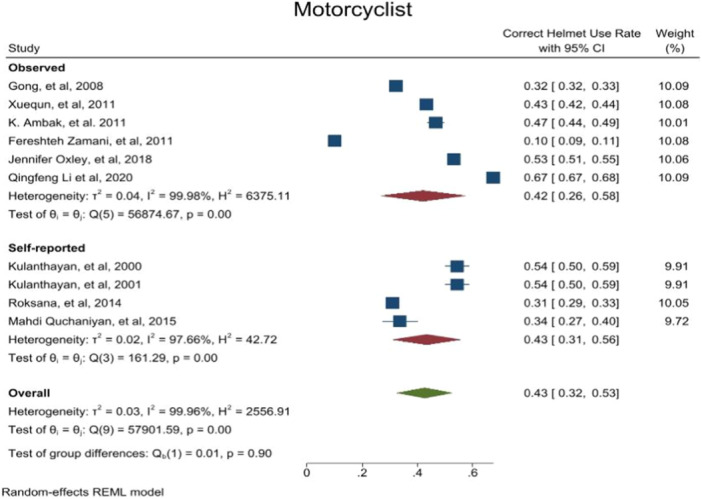
Pooled effect size of correct helmet use rate based on methods subgroups.

**FIGURE 5:**
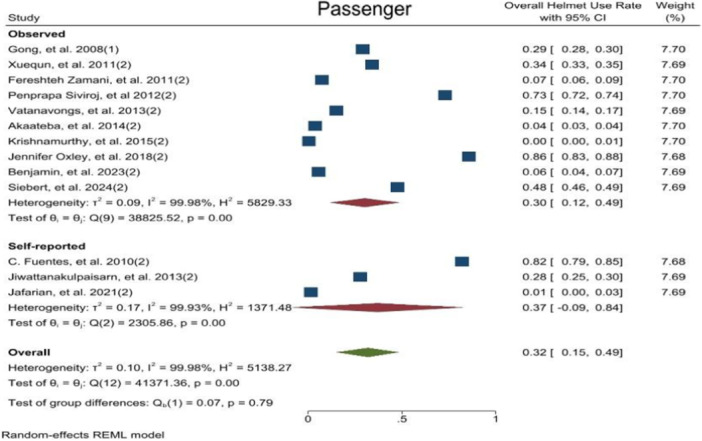
Pooled effect size of overall helmet use rate based on methods subgroups.

**FIGURE 6:**
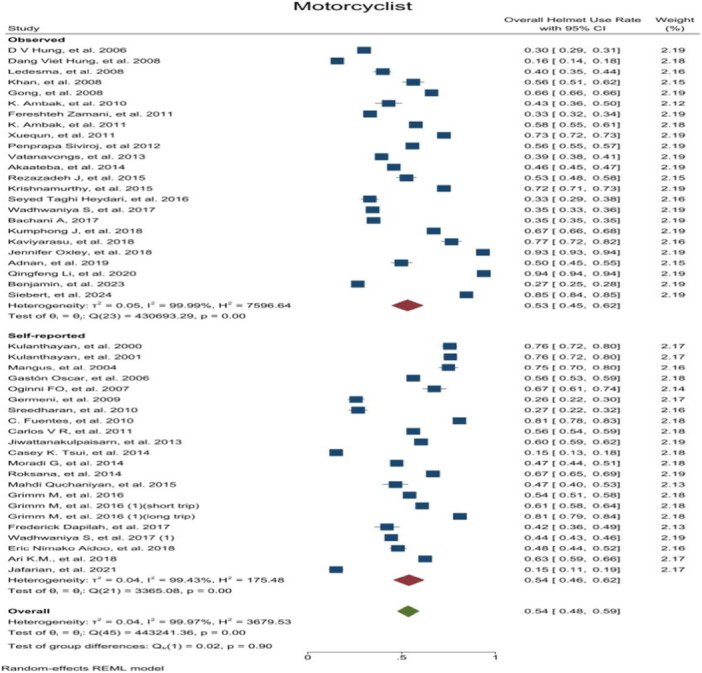
Pooled effect size of overall helmet use rate based on methods subgroups.

A meta‐analysis of the difference between the overall helmet use and correct helmet use was performed based on data collection methods (self‐reported/observed). The results disclosed no significant difference between two groups (*p*‐value > 0.05) (Figures 4, 5, 6).

### 3.4 Quality Appraisal Results

The average overall quality score of reporting was 27.7 (range = 0–36) in included studies. Generally, the reporting quality of articles was estimated as good (Appendix 4).

### 3.5 Determinants of Helmet Use in Motorcyclists

Content analysis was used to identify helmet use determinants/facilitators in 66 included studies. Totally 96 determinants in eight categories of psychological and cognitive factors, socio‐demographics, travel characteristics, road characteristics, motorcycle related issues, time/weather, law enforcement, and miscellaneous (Table 1).

**TABLE 1:** Determinate of helmet use in motorcyclists with frequency.

Psychological and cognitive (*n*)	Socio‐demographic	Travel characteristics	Road	Motorcycle	Miscellaneous	Time/weather	Law enforcement
Belief on helmet effects on reducing injury severity (17) Believe in law effectiveness (2) Riders with more motorcycle driving experience (8) Injury experience Belief on helmet mandatory law Having history of an unprotected motorcycle crash (4) Perceived more cues to action More self‐efficacy of riders The beliefs and positive influence of family, friends, and peers (9) Perceived vulnerability and severity of injury (2) Understanding the necessity, benefits, and importance of using helmets (3) Perception of social norms (2) Higher exposure to road safety awareness campaign/media (2) Positive implicit attitude Risk perception Perceived behavioral control while helmet usage (2) Risk‐averse drivers Drivers who show a higher awareness of road risks Not having sought information about protective clothing (2) Use of helmets by others Fear of death Fear of being fined	Individuals with:	The length of the trip (greater than 10 km) Traveling at 1 km to 10 km Distance traveled per trip Distance and time of travel	Traveling on a mandated road Riding on secondary street Riding on primary or secondary roads Compulsory roads Driving in housing estates area compared to countryside Riding on city streets Riding in central area of city (6) Riding in main streets Number of lane (2 lanes) Expos to traffic both in the city and on the road	Higher motorcycle class (2) Motorcycle with more than 250 cc volume (3) Riding a registered motorcycle Motorcycle brand Cruiser riders	Convenience of helmet use Being a rider rather than passengers (4) Racing (2) Safety and protection from pollution/dust (3) The presence of a helmeted passenger Using motorcycle for business (2) Driving while drunk Lack of health insurance Having a passenger (2) Traveling for work/school Ownership of a helmet Carrying less passengers Imitation Being the head of a household/family High cost of treatment due to the effects of the accident Imposing financial burden on the government Frequently using motor Being a pillion passenger	Riding in a rainy weather (4) Riding in morning period Riding during daytime hours (5) Riding during weekdays (2) Riding during weekends Weekend compared with weekday Weather conditions (Cold)	Having license (6) Passing formal training course Law requirement (6) Caught for non‐helmet use (3) Police controls and higher fine (14) Legal riders
	Higher education (14) Higher socio‐economic class Higher income (4) Younger age (5) Older age (21) Being married (4) Being male (9) Being female (9) Gender (2) Younger compared with older female Being city rider vs. rural rider (3) Being resident of a non‐slum area						

### 3.6 Barrier to Helmet Use in Motorcyclists

Psychological and cognitive factors were also identified as the most frequent items as barriers of helmet using among motorcyclists. Identified barriers were classified into six categories, including psychological and cognitive factors, socio‐demographics, travel/road characteristics, helmet related issues, time/weather, and miscellaneous (Table 2).

**TABLE 2:** Barrier to helmet use in motorcyclists with frequency.

Psychological and cognitive (*n*)	Socio‐demographic (*n*)	Travel/road characteristics (*n*)	Helmet (*n*)	Miscellaneous (*n*)	Time/weather (*n*)
Lower risk perception (2) Lack of appropriate information on helmet use High confidence of riders Fatalism (predetermination of death, disability of helmet for changing the fate, ignorance of human agency, belief in God's will on death time, and narration of death stories of motorcyclists who used helmet) Social relationship barriers (barrier to greeting, face‐to‐face relation, and tool of covering identity of motorcyclist) Peer group pressure and negative labeling (3) Belief on helmet non‐beneficiary on slow driving (2) Not enjoy when wearing a helmet (attitude) Low awareness of the danger of non‐helmet use Negative attitude toward helmet usage (2) Belief about helmet need for long trips only Uninvited attention from others Other motor riders' non‐use of helmet Forgetfulness	Riders in low socio‐economic status Rider with no or lower income use lower than rider with middle or upper income Female drivers and passengers less than male Young riders compared with the elderly	Traveling in a short distance (3) Driving in local road (2) Having lower number of trips	High helmet cost (4) Not owning a helmet (3) Messing up the appearance (a barrier to self‐presentation, messing up hair, covering the face) (3) Disturbance in hearing and vision (reduction of surrounding noise such as vehicle, car horns, etc., misrecognition of the car behind, restriction of seeing the left and right sides) (9) Barrier to normal breathing Barrier to enjoying pleasant weather Heaviness and superfluity of helmet (restriction of neck rotation, pain in neck and head, and inconveniency of carrying the helmet) (8) Discomfort (suffocation, it feels hot, and it feels heavy on the head) (13) Low quality of helmets (2) Concerns about physical appearance	Child pillion passengers Occupation: motorcyclists working in the agricultural sector, students, unemployed Being a passenger Alcohol use effect (2) Use a mobile phone while riding Rider compared with pillion High number of passengers	Sunny and likely, warm days Riding in weekends Riding in morning, at mid‐day and in the afternoon as compared to early morning Riding at night

## 4 Discussion

This systematic review and meta‐analysis were conducted to estimate the helmets use rate in motorcyclists and passengers and its determinants and barriers based on the results of 66 studies on approximately the one million people. Meta‐analysis results showed that OHUR in motorcyclists and passengers was 0.54 [0.48–0.59 with 95 CI, I^2^ = 99.9] and 0.32 [0.15–0.49 with 95 CI, I^2^ = 99.9], respectively. Also, CHUR in motorcyclists was estimated to be 0.43 [0.32–0.53 with 95 CI, I^2^ = 99.9] and 0.18 [0.12–0.24 with 95 CI, I^2^ = 9] in passengers. According to the content analysis, 86 unique determinates and 42 unique barriers to helmet use were identified. Psychological and cognitive factors were the most frequent items introduced both as determinant and barrier for helmet using. Estimated overall helmet using rate by motorcyclists was higher than the rate reported by pervious individual studies and in some cases was lower. This diversity could be related with cultural differences of various study settings, mandatory helmet low and socio‐economic situation. The results of this study provide an overall global estimation which could be referred as benchmarking rate.

Psychological and cognitive factors were the most frequent determinants and barriers of helmet use. Driver positive attitude and beliefs about helmet safety effects, risk perception and knowledge on protective devices were highlighted in this category. Literature showed that having positive attitude and beliefs about safety effects and protective rule of helmet promotes drivers behavior [18, 19]. Some studies in Iran showed that drivers power on controlling driving behaviors and having high self‐confidence had a significant relationship with intend to use the helmet [20, 21]. A recent study in Ethiopia revealed that high perceived susceptibility to crash had a significant association with helmet use among motorcyclists [22]. It was reported that drivers awareness on the helmet importance and safety effects, have more influence than mandating [23]. These sentences emphasize the importance of motorcyclists' mindfulness about helmet and its effects on employing safe behavior. As some previous studies reported that motorcyclists with crash experience are more likely to use helmet than others [24, 25].

Socio‐economic and demographic variables were also identified both as facilitators and barriers. Among the identified factors, having higher level of education, older age, and gender had the highest frequency. Studies in Nigeria and Australia, showed no significant relationship between motorcyclists socio‐economic status, occupation, education, and using helmet [10, 26]. This was inconsistent with result of most studies indicating significant association between socio‐economic status and educational level with motorcyclists' compliance of helmet use [12, 25, 27, 28, 29]. It is evident that good socio‐economic status will help individuals to prioritize their own safety. Moreover, higher educational level brings safety knowledge which creates positive attitude about safety effects of helmet and this guide motorcyclists to use helmets [20, 28, 30]. Standard helmet high price was introduced as an obstacle for using which is highlighted with poor socio‐economic situation of motorcyclists [31, 32]. Governmental or private supportive policies on helmet using and employing strategies to enhance public knowledge and attitude about helmet safety effects could be selected as encouraging policies.

Controversies results were reported in relation to the gender in the included studies as some reported that women use helmet more than men and vice versa [18, 27, 28, 33, 34, 35, 36, 37]. Gender effect may be issued as a cultural factor as in some countries such as Iran, only men use motorcycle. Generally, it was revealed that women had more adherence of helmet using law [38, 39]. Motorcyclist age was another variable which affect their helmet use behavior. It was reported that older drivers use helmet more than young people [4, 20, 25, 28, 31, 37] while in some other studies it was vice versa. Study by Faryabi et al. in Kerman, Iran, had reported that helmet use increases by age increase [40]. A recent study results revealed that each year increase in motorcyclists age was related with 0%/4% increase in helmet use [41]. It was mentioned in literature that youth (17–25 years) have less information about motorcycle safety, as well as improper attitudes toward the use of protective equipment, such as helmets, compared with adults [10, 11, 24]. Peer and family influence is the way that could be used to improve youth safety behavior. It was reported in previous studies that young motorcyclists employ safer behavior when they friends or family approve their behavior [24, 42].

It was revealed that long‐distance driving, riding at the roads inside the city compared to outside and riding in primary roads encourage helmet using. Surprisingly, it was reported that more than 85% of motorcyclists believe that the use of the helmet is not necessary, at low speeds, and short‐term travel and the roads within the city (district roads) [43]. A study in USA showed that traveling over long distances (more than 2 km) compared to short distances increase proper use of a helmet approximately about 6.5 times [18]. Similar results were also reported in studies from other countries [19, 25, 29, 33, 39]. Safe behavior of motorcyclists in all types of roads could be addressed through using traffic signs and enforcement. Using helmet in short‐distance travels and city inside are seen to be more related with motorcyclists' perception and this might be adapted through community awareness and risk perception arising.

Helmet characteristics, such as weight, cost, and discomfort (visual/audial problems) were discovered as barriers to be used. These items were raised as helmet using barriers in a systematic review among Iranian motorcyclists [2]. Studies from other countries were also reported that visual and audial limitation and blockage and heaviness of the helmet are among the barriers [8, 22, 35, 44]. In addition, literature showed that helmet use declines in the warm months [24, 44] and on the contrary, it increases in rainy conditions [36]. This refers to the helmets thermal dysregulation which was introduced as a barrier. Developing a helmet ergonomically with gender and age adjustments could address these obstacles. The government and nongovernmental organization dealing with road traffic prevention should advocate and ensure that standard helmets are made available at affordable price to both motorcyclist and passenger.

Motorcycle brand and motor high volume were reported in studies as helmet use facilitators. Motor riders, especially youth, are interested in maintaining their social class and prestige through using known and strong motors. This will be an opportunity to promote helmet using among youth who are under the influence of their peers.

Implementing helmet law and enforcement was discussed as another factor which forced motorcyclists to use helmet. Studies have approved the helmet law enforcement effects on increasing helmet usage [13] and injury reduction but it is criticized by researchers and suggested to be implemented align with other educational and cultural interventions [45]. Providing comprehensive interventions addressing helmet use from various aspects should be employed to increase effectiveness.

A methodological consideration in helmet use research was the potential for bias associated with the data collection methods, particularly social desirability in self‐reported studies. Our subgroup meta‐analysis revealed no statistically significant difference between observed and self‐reported studies across helmet use rate as outcome. Contrary to common, this finding suggest that self‐reported data in this field can provide estimates similar to observational studies and may not be systematically inflated.

## 5 Conclusions

Although the overall helmets use in motorcycles was about 50%, the correct use rate was almost half. The overall helmets use in self‐reported studies was higher than in observed studies, with no significant difference. Helmet using facilitators and barriers were identified and categorized in eight categories, including psychological and cognitive, socio‐demographic, travel characteristics, road characteristics, motorcycle, time/weather, law enforcement, and others. Continuous community‐based education on the importance of helmet use should be addressed through media and social campaigns to improve positive attitude and mindfulness.

### 5.1 Limitations

A very high level of heterogeneity was found among the included studies. This was expected, as the current meta‐analysis combined helmet use rates from various countries with different contexts, cultures, population, road conditions, and enforcements. In meta‐analyzes of prevalence, high levels of heterogeneity are to be expected and do not mean that the meta‐analysis is flawed but rather that the random effects model is used to estimate the average distribution of helmet use rates. Thus, the pooled estimates in the current study should be viewed as global average levels.

Another limitation is that even though the subgroup meta‐analysis revealed no statistically significant difference between the rates of observed and self‐reported helmet use, this result should be treated with caution. Ideally, a comparison between these two techniques would require studies that employ both techniques in the same population and during the same time period.

## Author Contributions

**Ramin Rezapour:** design, supervision, literature search, methodology, data extraction, analysis, writing – review and editing, writing – original draft, final approval. **Reza Ghorbani:** investigation, methodology, validation, writing – review and editing, writing – original draft, final approval. **Naser Derakhshani:** investigation, methodology, validation, writing – review and editing, writing – original draft, data curation. **Leili Abedi:** investigation, litrature screening, methodology, analysis, validation, writing – review and editing, writing – original draft, final approval. **Mahdi Sayyadzadeh:** investigation, methodology, validation, writing – review and editing, writing – original draft, final approval. **Mohammad Saadati:** design, supervision, literature search and screening, methodology, data extraction, analysis, writing – review and editing, writing – original draft, final approval.

## Ethics Statement

This study protocol was approved by the Research Ethics Committee of Tabriz University of Medical Science (ethical code: IR.TBZMED.VCR.REC.1399.213). Methods were performed in accordance with the relevant guidelines and regulations.

## Consent

The authors have nothing to report.

## Conflicts of Interest

The authors declare no conflicts of interest.

## Transparency Statement

The lead author Mohammad Saadati affirms that this manuscript is an honest, accurate, and transparent account of the study being reported; that no important aspects of the study have been omitted; and that any discrepancies from the study as planned (and, if relevant, registered) have been explained.

## Supporting information

Appendix 1.

Appendix 2.

Appendix 3.

Appendix 4.

## Data Availability

All data generated or analyzed during this study are included in this article.
