# Supplementary material for: Global Evidence on Helmet Use and Misuse: A Public Health Perspective on Prevalence, Determinants and Barriers
Source: Health Sci Rep. 2026 Mar 29;9(4):e72078. doi: 10.1002/hsr2.72078 (PMC13087616; doi:10.1002/hsr2.72078)
Supplement: Supplementary file 1 — Appendix 1. [file HSR2-9-e72078-s004.docx]

| Complete search strategy for PubMed databases | |
| --- | --- |
| **Set** | **Strategy** |
| #1 | (((Helmet[Title/Abstract])) OR (Helmet use[Title/Abstract])) AND (((((predictor[Title/Abstract]) OR(determinant[Title/Abstract])) OR (Barrier[Title/Abstract])) OR (affecting factor[Title/Abstract])) OR (facilitator[Title/Abstract])) |
| *****Filters activated: English | |
