# Supplementary material for: Global Evidence on Helmet Use and Misuse: A Public Health Perspective on Prevalence, Determinants and Barriers
Source: Health Sci Rep. 2026 Mar 29;9(4):e72078. doi: 10.1002/hsr2.72078 (PMC13087616; doi:10.1002/hsr2.72078)
Supplement: Supplementary file 2 — Appendix 2. [file HSR2-9-e72078-s003.docx]

Appendix 2: Epidemiological characteristics of helmet use among motorcyclists

| **No** | **Authors; Year** | **country** | **Study design** | **Barriers to wearing helmet** | **Determinants of using helmet** |
| --- | --- | --- | --- | --- | --- |
| 1 | Dang Viet Hung, et al; 2008(1) | Vietnam | Cross-sectional |  | - Older aged motorcyclists - Motorcycle drivers (rather than passengers) - The length of the trip (greater than 10 km) - Travelling on a mandated road - Having a university-level education |
| 2 | Liz de Rome, et al; 2010(2) | Australia | Cross-sectional |  | - Not having sought information about protective clothing - Not believing in its injury reduction value - Youth (17-25 year) - The type of motorcycle |
| 3 | Li-Ping Li, et al; 2008(3) | - | Review |  | - Male gender - Younger age - Riding on secondary streets - Riding during weekends - Cope with police - Prevent/reduce head injury |
| 4 | Konstantina Gkritza, et al; 2009(4) | United states | Cross-sectional | - sunny and likely, warm days - lower risk perception - traveling shorter distances | - Riding on primary or secondary roads - The presence of a helmeted passenger |
| 5 | K. Ambak, et al; 2010(5) | Malaysia | Cross-sectional | - | - Female riders, - Higher educated riders, - Full shell helmet users, - Travelling at a distance of 1 km to 10 km, - Riders having good practice - Riders in the town area |
| 6 | D V Hung, et al; 2006(6) | Vietnam | Population-based observational surveys. | - | - Male drivers - Adult drivers compared with young drivers - Compulsory roads |
| 7 | Ambak K, et al; 2011(7) | Malaysia | Cross-sectional |  | - Male drivers - Driving in housing estates area compared to countryside - Attitude about preventing head injuries |
| 8 | Rubén Daniel Ledesma, et al; 2008(8) | Argentina | Cross-sectional |  | - Male drivers - Riding in central area of the city - Riding in a rainy weather - Riding Motorcycle + 250 cc |
| 9 | Aida Bianco, et al; 2005(9) | Italy | Cross-sectional | - Discomfort - Not owning a helmet | - Adolescents with at least one family member who wore a helmet - Youths who agreed that helmet use should be mandatory - Those who did not experience an crash in the previous year |
| 10 | Olakulehin O.A, et al; 2015(10) | Nigeria | Cross-sectional |  | - Riding in day compared to night - Male riders - Older age riders |
| 11 | Imran Khan, et al; 2008(11) | Pakistan | Cross-sectional | - helmet’s bulk and inconvenience - physical - discomfort from heat - limited vision | - Higher education - Higher personal income - Protection - Riders view to protect from injury - Protection from dust - History of an unprotected motorcycle crash - Knowledge of someone else getting hurt in an - Unprotected motorcycle crash |
| 12 | S Kulanthayan, et al; 2000(12) | Malaysia | Cross-sectional | - | - Older riders - Female riders - Higher education - Having full license - More riding experience - Riders without prior crash involvement |
| 13 | R S Mangus, et al; 2004(13) | USA | Cross-sectional | - history of either injury(low use) | - Older riders - Higher education - Urban background compared to rural |
| 14 | Yu Xuequn, et al; 2011 (14) | China | Cross-sectional |  | - City riders compared to rural riders - Male gender - Riding on city streets - Being a driver compared to passengers - Carrying less passengers - Riding a registered motorcycle |
| 15 | Millicent Awialie Akaateba, et al; 2014(15) | Ghana | Cross-sectional |  | - Female gender - Weekdays - Morning period - Riding in locations within the Central Business District |
| 16 | S. Kijlanthayan, et al;  2001(16) | Malaysia | Cross-sectional |  | - Riding in town areas - Older riders - Female riders - Distance traveled per trip - Enforcement Prediction - Riding experience |
| 17 | Teamur Aghamolaei, et al; 2011(17) | Iran | Cross-sectional |  | - Perceived more behavioral control - Perceived more cues to action - More self-efficacy - Fewer barriers - Age - Education |
| 18 | E Germeni, et al; 2009(18) | Greece | Observational | - Peer pressure; - lack of appropriate information on helmet use; - high helmet cost; - lack of convenience; vision and hearing disturbance while driving, - style reasons(Beauty and style reasons) | - Protection in the case of road crash - Avoiding tickets from traffic police |
| 19 | Ruben D. Ledesma, et al; 2014(19) | Argentina | Observational | - weekends | - Male drivers - Riding in central area of the city - Riding in a rainy weather - Riding Motorcycle + 250 cc |
| 20 | Gong-li Li, et al; 2008(20) | China | Cross-sectional |  | - Riding in main streets - Riding during daytime hours - Riding during weekdays - Prevent or alleviate head injury |
| 21 | Roksana Mirkazemi,et al; 2014(21) | India | Observational |  | - Age older - Higher socio-economic class - Residents of non-slum areas - Widows - Belonging to - Extended family |
| 22 | Oginni FO, , et al; 2007(22) | Nigeria | Observational | - cost - Cross infection - number of myths |  |
| 23 | Khanh H. Pham, et al; 2008(23) | Vietnam | Observational |  | - Higher income - Older age |
| 24 | Vatanavongs, et al; 2013(24) | Thailand | Cross- sectional |  | - Gender(male) - Age group(Adult) - Number of lane (2 lans) - Location(segment) - Time of day(Day) |
| 25 | Casey K. Tsui, et al; 2014(25) | United States American | Observational |  | - Race - Motorcycle brand - Years of riding experience - Having taken a formal training course - Cruiser riders |
| 26 | Fereshteh Zamani, et al; 2011(26) | Iran | Mixed-method |  | - Encouragement or the positive influence of others - Being the head of the household/family - Perceived vulnerability and severity of crash related injury - Belief in helmet efficiency - Other perceived benefits of the helmet - Having direct and indirect experiences of traffic injury |
| 27 | Mahdi Quchaniyan Haqverdi, et al; 2015(27) | Iran | Cross sectional | - Feeling of heat - Decreasing vision and hearing | - Perception of social norms - Tendency to engagement in risky traffic behaviors - Understanding the necessity, importance of using helmets - Ease of use |
| 28 | Mehri Ali, et al; 2011 (28) | Iran | Review |  | - Attitude - Subjective norms - Perceived behavioral control |
| 29 | Javad Faryabi, et al; 2014(29) | Iran | Cross-sectional | - The heavyweight of helmet - Feeling of heat - Pain in the neck - Feeling of suffocation - Limitation of movement of the head and neck - Physical discomfort |  |
| 30 | Seyed Taghi Heydari, et al; 2016(30) | Iran | Cross-sectional |  | - Older age of rider - Married people compare with singles - Having more driving experience - Using motorcycle for business reasons - Having license |
| 31 | Ghobad Moradi, et al; 2014(31) | Iran | Surveillance data | - Low socio-economic status |  |
| 32 | Rezazadeh, et al; 2015(32) | Iran | Cross-sectional |  | - Married people compare with singles - Older age of rider - Higher education - Having license - Living in city compare with village |
| 33 | A.M. Bachani, et al; 2016(33) | Kenya | Observational  (Inferential study) | - morning, at mid-day and in the afternoon as compared to early morning | - Weather condition (light rain associated with correct helmet use compared to dry period) |
|  |  |  | Survey  (KAP Study) | - inconvenient/ uncomfortable - I forget sometimes - The helmet is broken - not having a helmet - driving in local road - I am a highly skilled driver and helmet is unnecessary | - It's required by law - Police can fine me if I don't - It can save my life |
| 34 | Jennifer Oxley, et al; 2017(34) | Malaysia | Observational | - child pillion passengers | - adults and pillion passenger |
| 35 | Frederick , et al; 2017(35) | Ghana | Descriptive:  Questionnaire & Interview | - Ownership of motorcycle - Occupation: motorcyclists working in the agricultural sector, students, unemployed and motorcyclist working in the commerce sector | - Old rider (30-39) - Occupation: motorcyclists working in the services sector. |
| 36 | Sadegh Ghasemzadeh , et al; 2017(36) | Iran | Cross sectional | - rider with no or lower income use lower than rider with middle or upper income | - Fined by police for not wearing helmet - Perceived behavioral control - Social norms |
| 37 | Aliasghar Maghsoudi, et al; 2017(37) | Iran | Qualitative - interview | **Fatalism:**   - Predetermination of death - Disability of helmet for changing the fate - Ignorance of human agency - Belief in God’s will on death time - Narration of death stories of motorcyclists who used helmet   **Barriers to social relationship:**   - Barrier to greeting - Barrier to face-to-face relation - Barrier to kissing and hugging - Tool of covering identity of motorcyclist   **Peer group pressure and negative labelling:**   - Prevalence of not using helmet among peer - Dissatisfaction of using helmet by others - Being ridiculed or ostracized - Strategy of labelling - ‘coward’, ‘old man’, ‘conservative’, ‘goody-goody’, ‘positive guys’   **Messing up the appearance:**   - A barrier to self-presentation - Messing up hair - Covering the face   **Disturbance in hearing and vision**   - Reduction of surrounding noise such as vehicle, car horns, etc.   – Misrecognition of the car behind  – Restriction of seeing the left and right sides  **Barrier to normal breathing**  – By producing heat and steam  – Breathing hard in summer  – Barrier to enjoying pleasant weather  **Heaviness and superfluity of helmet**  – Restriction of neck rotation  – Pain in neck and head  – Inconveniency of carrying the helmet |  |
| 38 | Krishnamurthy Karuppanagounder, et al; 2015(38) | India | Qualitative –  Observation and interview | - no need if drive slowly and carefully - need for long trips only - effects driving control - affects hearing - blocks vision - cause hair loos - cause neck pain - helmet costly | - To increase the safety - To get away from police - To escape from rain - To get protection from dust - Police checking - Helmet price moderate and low - Need strict enforcement to improve usage comfortable |
| 39 | Jayadevan Sreedharan, et al; 2009(39) | India | Cross-sectional |  | - Stringent legislative measures would promote the regular use of helmet by motorcyclists - Ignorance about the need for a safety helmet - Gender - Marital status - Driving while drunk - Use of alcohol - Attitude towards implementing legislative measures in using helmet |
| 40 | M L Ranney, et al; 2010(40) | USA | Web-based survey |  | - Gender - Education - Decreasing vision or hearing - Wear helmets because forced by law - Helmet use correlated more closely with attitudes than with norms |
| 41 | Rubén D. Ledesma, et al; 2015(41) | Argentina | Mixed method | - Feeling of discomfort | - Positive implicit attitude |
| 42 | RakhiDandona, G, et al; 2006(42) | India | Cross sectional |  | - Distance and time of travel - Discomfort (suffocation, it feels hot, and it feels heavy on the head) - Safety and protection from pollution |
| 43 | Carlos V. R, et al; 2011(43) | USA | Review |  | - Younger comparison to older females - Race - Alcohol intoxication - Riding as a passenger - Lack of health insurance |
| 44 | Concepci Fuentes, et al; 2010(44) | Spain | Cross sectional |  | - Increased age - Belief in helmet effectiveness - Passengers believe - Frequency of driving/riding as a passenger one motorcycle - The belief to the effectiveness of helmet-use - The belief that friends and relatives always use a helmet |
| 45 | M. Papadakaki, et al; 2013(45) | Greece | Cross sectional |  | - **Trip destination**   (frequently ‘‘when going home’’ and ‘‘when going to work or school’’---less frequently ‘‘when travelling’’ and ‘‘while being at work/school’’)   - **Places** - **Emotional conditions**   (‘‘happy or excited’’ and less frequently when they felt ‘‘competitive or joyful’’ and when they were ‘‘flirting with the opposite sex’’)   - Frequency of motorcycle riding - **Different seasons**   (less frequently during spring compared to the other seasons)   - **Socio-demographic characteristics** - Gender - Years of education - Consumption alcohol - Time of day where riding is taking place - **Facilitators and barriers** - Imitation - Experience - Self-protection - Environment - Regulation - Discomfort - Underestimation of danger |
| 46 | Ahmed, Mohamad Baharin; 2013(46) | Malaysia | Cross sectional |  | - Short distance - Attitude - Subjective norm (family or close friend) - Descriptive norm (other people) - Type of road |
| 47 | Nur Sabahiah A.S, et al; 2011(47) | Malaysia | Cross sectional | - Not enjoy wearing a helmet - Felt wearing a helmet was hard | - Gender - Perception of danger - Fear of being caught - Moral obligation - Perceived behavior control - Age: Younger motorcyclists are more likely than older motorcyclists to not wear a helmet - Motorcyclists that had received at least one ticket. |
| 48 | Gastón Oscar Babi, et al; 2006(48) | Indonesia | Cross-sectional |  | - Increased age - Road exposure - Expos to traffic in the City and on the road - Educational level - Community size - The average family income of the community |
| 49 | Piyapong Jiwattanakulpaisarn, et al; 2013(49) | Thailand | Cross sectional |  | - Awareness of helmet law enforcement - Checkpoints to take place - Frequently observing the police’s checkpoints - Perceiving the high risk of being caught for non-helmet use - Absence of knowledge on the compulsory helmet law for passengers. - Law was not enforced by the police. - Age:(older and male) - Frequency use of the motorcycle - Female passengers wore a helmet less frequently. - Gender - age - Riding frequency - Gender: (male drivers in the older age) - Perception of police checkpoints for helmet usage |
| 50 | Siviroj, Penprapa  Peltzer, et al; 2012(50) | Thailand | Cross-sectional | - Helmets less used in low and middle income countries. - during the Songkran festival - during the day - on roads out of town - on highways - history of crash - low awareness of the danger of non-helmet use - caught for non-helmet use | - Higher exposure to road safety awareness campaign - Motorcyclist who have no passenger |
| 51 | Nur Sabahiah A.S, et al; 2011(51) | Malaysia | Cross sectional | - Motorcyclists in the paved shoulder felt hard to wear a helmet while riding the motorcycle. | - Riding in the paved shoulder compared to the exclusive and inclusive lane |
| 52 | Kris Brijs, et al; 2014(52) | Cambodia | Cross-sectional | - uncomfortable when it is hot - not fashionable - difficult to hear and see traffic | - Believing that helmet protects from head injuries - Believing that helmet protects from dust/wind/rain - Believing that helmet protects from getting into trouble with police - Believing that helmet better protects from serious head injury - The subjective estimation of personal control over specific situational factors - Perceived susceptibility - Personal norm - Behavioral intentions. |
| 53 | S. Wadhwaniya, et al; 2017)(53) | India | KAP Study: Interview | - Male gender - youth (24 years) - a lower level of education - non-ownership of helmet |  |
| 54 | Michael Grimm, et al; 2016(54) | India | Data set study | - | - Risk-averse drivers more than others - Trained drivers - Drivers who show a higher awareness of road risks |
| 55 | Muhammad Adnan 2019(55) | - | Cross-sectional | - having lesser number of trips | - Have a valid driver's license - Have more experience of motorcycling - have higher monthly income - Older aged motorcyclists |
| 56 | Christopher A 2019 (56) | Thailand | Systematic review | - alcohol use effect - Female drivers and passengers less than male | - Older aged motorcyclists - traffic injuries related to motorcycles - Not having sought information about protective clothing |
| 57 | Ari K.M. Tarigan 2018 (57) | Malaysia | Review | - Not use of helmet by others | - Older aged motorcyclists - motorcycle class - ticketing for a traffic violation - experiences of having traffic crashes |
| 58 | Kumphong J 2018 (58) | Thailand | Observational |  | - Older aged motorcyclists - motorcycle class - time of day - The riding status (Riders relative to passengers.) - Existence of police booths - number of passengers - Legal riders. |
| 59 | Eric Nimako Aidoo 2018 (59) | Ghana | Cross-sectional | - uncomfortable using a helmet - Short distance - Forgetfulness - blocked the vision or hearing - Not having a helmet | - Female gender - Married - Education - Motorcycle license - frequently use |
| 60 | Li Q, Adetunji, et al; 2020 (60) | Vietnam | Cross-sectional |  | - Correct helmet use is higher for adults than for children. |
| 61 | Vahid Ranaei, et al; 2021 (61) | Iran | Qualitative - interview | - High price of helmets - Low quality of helmets - To be ridiculed - Use a mobile phone while riding - Don't really believe in helmets | - High cost of treatment due to the effects of the accident - Imposing financial burden on the government - Family expenses provision - Family support - The value of health and welfare - Commitment to family - Use of helmets by others - Seeing the experiences of other people affected by not wearing a helmet is effective - Injury experience - Fear of death - Fear of being fined - Believe in law effectiveness - Vehicle detention |
| 62 | Saleh Jafarian, et al; 2021 (62) | Iran | Cross-sectional |  | - Age more than 35 years - High education - Having a driving license - Protection against injuries in accidents |
| 63 | Akuh R, et al; 2023 (63) | Ghana | Cross-sectional |  | - Perceived safety of the helmet - Weather conditions - Convenience of helmet use |
| 64 | Khan UR, et al; 2023 (64) | Pakistan | Qualitative – Focus discussion | - Discomfort in wearing a helmet - Uninvited attention from others - Concerns about physical appearance - Substandard quality and design of helmets | - Awareness generation through media - Complementary distribution of helmets - Strict law enforcement in the form of fines - The influence of religious leaders regarding social norms and cultural barriers |
| 65 | Benjamin, et al; 2023 (65) | Ghana | Cross-sectional | - Local roads compared with highways - Young riders compared with the elderly - Rider compared with pillion. | - Day compared with the night, - Weekend compared with weekday - Males compared with females |
| 66 | Siebert FW, et al; 2024 (66) | Madagascar | Cross-sectional | - High number of riders per motorcycle | - Drivers more than passengers - Female more than male |

**References**

1. Hung DV, Stevenson MR, Ivers RQ. Barriers to, and factors associated, with observed motorcycle helmet use in Vietnam. Accident Analysis & Prevention. 2008;40(4):1627-33.

2. de Rome L, Ivers R, Haworth N, Heritier S, Du W, Fitzharris M. Novice riders and the predictors of riding without motorcycle protective clothing. Accident Analysis & Prevention. 2011;43(3):1095-103.

3. Liu BC, Ivers R, Norton R, Boufous S, Blows S, Lo SK. Helmets for preventing injury in motorcycle riders. The Cochrane database of systematic reviews. 2008(1):Cd004333.

4. Gkritza K. Modeling motorcycle helmet use in Iowa: Evidence from six roadside observational surveys. Accident Analysis & Prevention. 2009;41(3):479-84.

5. Ambak K, Hashim H, Yusoff L, David B. An Evaluation on the Compliance to Safety Helmet Usage among Motorcyclists in Batu Pahat, Johor. International Journal of Integrated Engineering (Issue on Civil and Environmental Engineering). 2010;2(2):45-51.

6. Hung DV, Stevenson MR, Ivers RQ. Prevalence of helmet use among motorcycle riders in Vietnam. Injury Prevention. 2006;12(6):409-13.

7. Ambak K, Ismail R, Rahmat RAO, Shokri F. Do Malaysian motorcyclists concern to safety helmet usage: A Cross-sectional survey. J Appl Sci. 2011;11(3):555-60.

8. Ledesma R D, Peltzer R I. Helmet Use Among Motorcyclists: Observational Study in the City of Mar del Plata, Argentina. Rev Saude Publica 2008;42(1):143-5.

9. Bianco A, Trani F, Santoro G, Angelillo IF. Adolescents’ attitudes and behaviour towards motorcycle helmet use in Italy. European Journal of Pediatrics. 2005;164(4):207-11.

10. Olakulehin O A, Adeomi A A, Babalola O R, Olanipekun O O, Ilori O S. Helmet use among motorcycle riders Insemi-Urban communities in Southwestern Nigeria Journal of Medicine and Medical Sciences. 2015;6(3):35-9.

11. Khan I, Khan A, Aziz F, Islam M, Shafqat S. Factors associated with helmet use among motorcycle users in Karachi, Pakistan. Academic emergency medicine : official journal of the Society for Academic Emergency Medicine. 2008;15(4):384-7.

12. Kulanthayan S, Umar RS, Hariza HA, Nasir MT, Harwant S. Compliance of proper safety helmet usage in motorcyclists. The Medical journal of Malaysia. 2000;55(1):40-4.

13. Mangus RS, Simons CJ, Jacobson LE, Streib EW, Gomez GA. Current helmet and protective equipment usage among previously injured ATV and motorcycle riders. Injury prevention : journal of the International Society for Child and Adolescent Injury Prevention. 2004;10(1):56-8.

14. Xuequn Y, Ke L, Ivers R, Du W, Senserrick T. Prevalence rates of helmet use among motorcycle riders in a developed region in China. Accident; analysis and prevention. 2011;43(1):214-9.

15. Akaateba MA, Amoh-Gyimah R, Yakubu I. A Cross-sectional observational study of helmet use among motorcyclists in Wa, Ghana. Accident; analysis and prevention. 2014;64:18-22.

16. Kulanthayan S, Radin Umar R S, Ahmad Hariza H, Mohd Nasir MT. Modeling of Compliance Behavior of Motorcyclists to Proper Usage of Safety Helmets in Malaysia. Journal of Crash Prevention and Injury Control. 2001;2(3):239-46.

17. Aghamolaei T, Tavafian SS, Madani A. Prediction of Helmet Use Among Iranian Motorcycle Drivers: An Application of the Health Belief Model and the Theory of Planned Behavior. Traffic Injury Prevention. 2011;12(3):239-43.

18. Germeni E, Lionis C, Davou B, Petridou ET. Understanding reasons for non-compliance in motorcycle helmet use among adolescents in Greece. Injury prevention : journal of the International Society for Child and Adolescent Injury Prevention. 2009;15(1):19-23.

19. Ledesma RD, Peltzer RI. Helmet use among motorcyclists: observational study in the city of Mar del Plata, Argentina. Revista de Saúde Pública. 2008;42(1):143-5.

20. Li GL, Li LP, Cai QE. Motorcycle helmet use in Southern China: an observational study. Traffic Inj Prev. 2008;9(2):125-8.

21. Mirkazemi R, Kar A. Socio-economic determinants of helmet-wearing behaviour in Pune city, India. International journal of injury control and safety promotion. 2014;21(4):376-81.

22. Oginni FO, Ugboko VI, Adewole RA. Knowledge, attitude, and practice of Nigerian commercial motorcyclists in the use of crash helmet and other safety measures. Traffic Inj Prev. 2007;8(2):137-41.

23. Pham KH, Le Thi QX, Petrie DJ, Adams J, Doran CM. Households' willingness to pay for a motorcycle helmet in Hanoi, Vietnam. Applied health economics and health policy. 2008;6(2-3):137-44.

24. Ratanavaraha V, Jomnonkwao S. Community participation and behavioral changes of helmet use in Thailand. Transport Policy. 2013;25:111-8.

25. Tsui CK, Rice TM, Pande S. Predictors of nonstandard helmet use among San Francisco Bay-area motorcyclists. Traffic Inj Prev. 2014;15(2):151-5.

26. Zamani-Alavijeh F, Bazargan M, Shafiei A, Bazargan-Hejazi S. The frequency and predictors of helmet use among Iranian motorcyclists: A quantitative and qualitative study. Accident Analysis & Prevention. 2011;43(4):1562-9.

27. Haqverdi MQ, Seyedabrishami S, Groeger JA. Identifying psychological and socio-economic factors affecting motorcycle helmet use. Accident Analysis & Prevention. 2015;85:102-10.

28. Ali M, Saeed MMS, Ali MM, Haidar N. Determinants of helmet use behaviour among employed motorcycle riders in Yazd, Iran based on theory of planned behaviour. Injury. 2011;42(9):864-9.

29. Faryabi J, Rajabi M, Alirezaee S. Evaluation of the use and reasons for not using a helmet by motorcyclists admitted to the emergency ward of shahid bahonar hospital in kerman. Archives of trauma research. 2014;3(3):e19122.

30. Heydari ST, Lankarani KB, Vossoughi M, Javanmardi K, Sarikhani Y, Mahjoor K, et al. The prevalence and effective factors of crash helmet usage among motorcyclists in Iran. Journal of injury & violence research. 2016;8(1):1-5.

31. Moradi G, Malekafzali Ardakani H, Majdzadeh R, Bidarpour F, Mohammad K, Holakouie-Naieni K. Socioeconomic Inequalities in Nonuse of Seatbelts in Cars and Helmets on Motorcycles among People Living in Kurdistan Province, Iran. Iranian journal of public health. 2014;43(9):1239-47.

32. Rezazadeh J, Rajabzadeh R, Jabbari S, Soliymani A, Emami O, Hosseini SH. Knowledge, attitude, and practice of the motorcyclists of Bojnourd regarding using helmets. Safety Promotion and Injury Prevention. 2015;2(4):303-12.

33. Bachani A, Hung Y, Mogere S, Akunga D, Nyamari J, Hyder AA. Helmet wearing in Kenya: prevalence, knowledge, attitude, practice and implications. Public health. 2017;144:S23-S31.

34. Oxley J, O'Hern S, Jamaludin A. An observational study of restraint and helmet wearing behaviour in Malaysia. Transportation Research Part F: Traffic Psychology and Behaviour. 2018;56:176-84.

35. Dapilah F, Guba BY, Owusu-Sekyere E. Motorcyclist characteristics and traffic behaviour in urban northern Ghana: Implications for road traffic accidents. Journal of Transport & Health. 2017;4:237-45.

36. Ghasemzadeh S, Babazadeh T, Allahverdipour H, Sadeghi-Bazargani H, Kouzekanani K. Cognitive-behavioral determinants of using helmet by motorcyclists in a rural community. Journal of Transport & Health. 2017;6:548-54.

37. Maghsoudi A, Boostani D, Rafeiee M. Investigation of the reasons for not using helmet among motorcyclists in Kerman, Iran. International journal of injury control and safety promotion. 2018;25(1):58-64.

38. Karuppanagounder K, Vijayan AV. Motorcycle helmet use in Calicut, India: User behaviors, attitudes, and perceptions. Traffic injury prevention. 2016;17(3):292-6.

39. Sreedharan J, Muttappallymyalil J, Divakaran B, Haran JC. Determinants of safety helmet use among motorcyclists in Kerala, India. Journal of injury and violence research. 2009;2(1):49-54.

40. Ranney ML, Mello MJ, Chai P, Baird J, Clark M. Determinants of motorcycle helmet use among recent graduates of a motorcycle training course. Injury Prevention. 2010;16(Suppl 1):A54-A.

41. Ledesma RD, Tosi J, Poó FM, Montes SA, López SS. Implicit attitudes and road safety behaviors. The helmet-use case. Accident Analysis & Prevention. 2015;79:190-7.

42. Dandona R, Kumar GA, Dandona L. Risky behavior of drivers of motorized two wheeled vehicles in India. Journal of safety research. 2006;37(2):149-58.

43. Brown CV, Hejl K, Bui E, Tips G, Coopwood B. Risk factors for riding and crashing a motorcycle unhelmeted. The Journal of emergency medicine. 2011;41(4):441-6.

44. Fuentes C, Gras ME, Font-Mayolas S, Bertran C, Sullman MJ, Ballester D. Expectations of efficacy, social influence and age as predictors of helmet-use in a sample of Spanish adolescents. Transportation research part F: traffic psychology and behaviour. 2010;13(5):289-96.

45. Papadakaki M, Tzamalouka G, Orsi C, Kritikos A, Morandi A, Gnardellis C, et al. Barriers and facilitators of helmet use in a Greek sample of motorcycle riders: Which evidence? Transportation research part F: traffic psychology and behaviour. 2013;18:189-98.

46. Ahmed MB, Ambak K, Raqib A, Sukor NS. Helmet usage among adolescents in rural road from the extended theory of planned behaviour. J Appl Sci. 2013;13(1):161.

47. A.S NS, Fujii S. Motorcyclists I – Helmet Usage And Speeding Behavior From A Psychological Perspective. Australian Journal of Basic and Applied Sciences. 2011;5(8):1115-20.

48. Babio GO, Daponte-Codina A. Factors associated with seatbelt, helmet, and child safety seat use in a Spanish high-risk injury area. Journal of Trauma and Acute Care Surgery. 2006;60(3):620-6.

49. Jiwattanakulpaisarn P, Kanitpong K, Ponboon S, Boontob N, Aniwattakulchai P, Samranjit S. Does law enforcement awareness affect motorcycle helmet use? Evidence from urban cities in Thailand. Global health promotion. 2013;20(3):14-24.

50. Siviroj P, Peltzer K, Pengpid S, Morarit S. Helmet use and associated factors among Thai motorcyclists during Songkran festival. International journal of environmental research and public health. 2012;9(9):3286-97.

51. Sukor NSA, Fujii S. The Effect of Psychological Factors towards Motorcyclists’ Risky Behaviours in Different Type of Motorcycle Lanes. International Journal of Humanities and Social Science. 2011;1 No. 9(Special Issue – July).

52. Brijs K, Brijs T, Sann S, Trinh TA, Wets G, Ruiter RA. Psychological determinants of motorcycle helmet use among young adults in Cambodia. Transportation research part F: traffic psychology and behaviour. 2014;26:273-90.

53. Wadhwaniya S, Gupta S, Mitra S, Tetali S, Josyula L, Gururaj G, et al. A comparison of observed and self-reported helmet use and associated factors among motorcyclists in Hyderabad city, India. Public health. 2017;144:S62-S9.

54. Grimm M, Treibich C. Why do some motorbike riders wear a helmet and others don’t? Evidence from Delhi, India. Transportation Research Part A: Policy and Practice. 2016;88:318-36.

55. Adnan M, Gazder U. Investigation of helmet use behavior of motorcyclists and effectiveness of enforcement campaign using CART approach. IATSS research. 2019;43(3):195-203.

56. German CA, Soontornmon K, Singkham P, Tanasugarn L, Thienmongkol R, Weeranakin N, et al. A systematic review on epidemiology and promotion of motorcycle helmet use in Thailand. Asia Pacific Journal of Public Health. 2019;31(5):384-95.

57. Tarigan AK, Sukor NSA. Consistent versus inconsistent behaviour of helmet use among urban motorcyclists in Malaysia. Safety science. 2018;109:324-32.

58. Kumphong J, Satiennam T, Satiennam W. The determinants of motorcyclists helmet use: Urban arterial road in Khon Kaen City, Thailand. Journal of safety research. 2018;67:93-7.

59. Nimako Aidoo E, Bawa S, Amoako-Yirenkyi C. Prevalence rate of helmet use among motorcycle riders in Kumasi, Ghana. Traffic injury prevention. 2018;19(8):856-9.

60. Li Q, Adetunji O, Pham CV, Tran NT, Chan E, Bachani AM. Helmet use among motorcycle riders in Ho Chi Minh City, Vietnam: results of a five-year repeated Cross-sectional study. Accident Analysis & Prevention. 2020;144:105642.

61. Ranaei V, Hosseini Z, Dadipoor S. Barriers to using a helmet among motorcyclist students: a qualitative study. Journal of injury and violence research. 2021;13(2):81.

62. Jafarian S, Ahmadi A, Amiri M, Biokani R, Abbaspour Z, Mahmoudzadeh M. The Prevalence of Helmet Use and Predictive Factors Among Motorcyclists in Shahrekord, Iran in 2018. Epidemiology and Health System Journal. 2021;8(1):9-13.

63. Akuh R, Donani M, Okyere S, Gyamfi EK. The impact of perceived safety, weather condition and convenience on motorcycle helmet use: The mediating role of traffic law enforcement and road safety education. IATSS Research. 2023.

64. Khan UR, Zia N, Khudadad U, Wright K, Sayed SA. Perceptions, barriers, and strategies regarding helmet use by female pillion riders in Pakistan: A qualitative study. Injury. 2023 Aug 1;54:110740.

65. Adjei BN, Nakua EK, Donkor P, Gyaase D, Alhassan MM, Amissah J, et al. Helmet utilisation and its associated factors among motorcyclists in northern Ghana: an analytical Cross-sectional survey. Injury prevention. 2024.

66. Siebert FW, Brambati F, Silva AL, Randrianarisoa J, Perego P. Gender disparities in observed motorcycle helmet use in Madagascar: female motorcyclists behave safer but have lower overall protection. Injury prevention. 2024.
