# Supplementary material for: Global Evidence on Helmet Use and Misuse: A Public Health Perspective on Prevalence, Determinants and Barriers
Source: Health Sci Rep. 2026 Mar 29;9(4):e72078. doi: 10.1002/hsr2.72078 (PMC13087616; doi:10.1002/hsr2.72078)
Supplement: Supplementary file 3 — Appendix 3. [file HSR2-9-e72078-s002.docx]

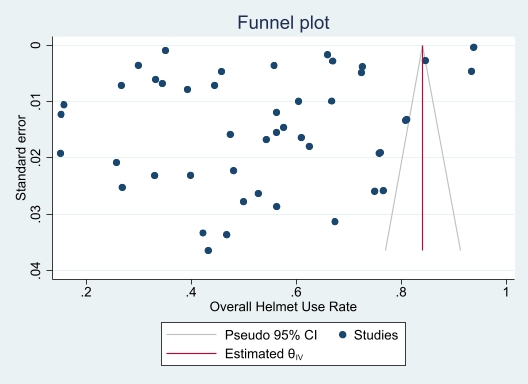


Appendix 3: Funnel Plots

A: Overall Helmet Use Rate - Motorcyclist


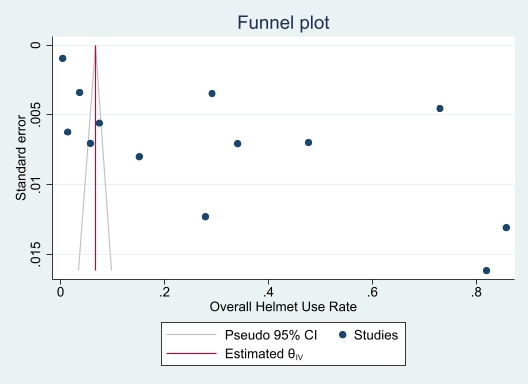


B: Overall Helmet Use Rate – Passenger


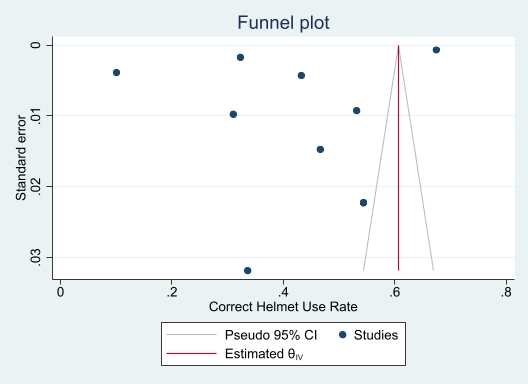


C: Correct Helmet Use Rate - Motorcyclist
