# Supplementary material for: Global Evidence on Helmet Use and Misuse: A Public Health Perspective on Prevalence, Determinants and Barriers
Source: Health Sci Rep. 2026 Mar 29;9(4):e72078. doi: 10.1002/hsr2.72078 (PMC13087616; doi:10.1002/hsr2.72078)
Supplement: Supplementary file 4 — Appendix 4. [file HSR2-9-e72078-s001.docx]

**Appendix 4: Result of quality assessment of included articles**

| **Sum** | **18** | **17** | **16** | **15** | **14** | **13** | **12** | **11** | **10** | **9** | **8** | **7** | **6** | **5** | **4** | **3** | **2** | **1** | **Questions****  **Studies** |
| --- | --- | --- | --- | --- | --- | --- | --- | --- | --- | --- | --- | --- | --- | --- | --- | --- | --- | --- | --- |
| 30 | 1 | 2 | 2 | 1 | 2 | 2 | 2 | 2 | 1 | 1 | 2 | 2 | 2 | 2 | 2 | 1 | 2 | 1 | Kulanthayan, et al. 2000 |
| 26 | 2 | 2 | 2 | 2 | 1 | 1 | 0 | 1 | 0 | 1 | 2 | 1 | 2 | 2 | 2 | 2 | 2 | 1 | Kulanthayan, et al. 2001 |
| 31 | 1 | 2 | 2 | 1 | 2 | 2 | 1 | 1 | 1 | 2 | 2 | 2 | 2 | 2 | 2 | 2 | 2 | 2 | Mangus, et al. 2004 |
| 29 | 2 | 2 | 1 | 2 | 1 | 1 | 1 | 1 | 2 | 2 | 2 | 1 | 2 | 2 | 2 | 2 | 2 | 1 | D V Hung, et al. 2006 |
| 32 | 1 | 1 | 2 | 1 | 2 | 2 | 2 | 2 | 2 | 2 | 2 | 2 | 2 | 2 | 2 | 2 | 2 | 1 | Gastón Oscar, et al. 2006 |
| 31 | 1 | 2 | 2 | 2 | 1 | 1 | 2 | 2 | 1 | 2 | 2 | 1 | 2 | 2 | 2 | 2 | 2 | 2 | Oginni FO, et al. 2007 |
| 30 | 2 | 2 | 1 | 1 | 2 | 2 | 1 | 2 | 1 | 2 | 1 | 2 | 2 | 2 | 2 | 2 | 2 | 1 | Dang Viet Hung, et al. 2008 |
| 31 | 2 | 2 | 2 | 2 | 2 | 2 | 1 | 2 | 0 | 1 | 2 | 1 | 2 | 2 | 2 | 2 | 2 | 2 | Ledesma, et al. 2008 |
| 31 | 1 | 2 | 2 | 1 | 2 | 2 | 2 | 2 | 0 | 2 | 2 | 2 | 2 | 2 | 2 | 2 | 2 | 1 | Khan, et al. 2008 |
| 29 | 1 | 1 | 2 | 2 | 1 | 1 | 2 | 2 | 2 | 2 | 1 | 1 | 2 | 2 | 2 | 2 | 2 | 1 | Gong, et al. 2008 |
| 31 | 0 | 2 | 2 | 2 | 2 | 1 | 2 | 2 | 1 | 2 | 2 | 2 | 2 | 2 | 2 | 2 | 2 | 1 | Germeni, et al. 2009 |
| 26 | 0 | 1 | 2 | 1 | 1 | 1 | 2 | 1 | 1 | 1 | 2 | 1 | 2 | 2 | 2 | 2 | 2 | 2 | K. Ambak, et al. 2010 |
| 30 | 2 | 1 | 2 | 1 | 1 | 1 | 2 | 2 | 2 | 1 | 2 | 1 | 2 | 2 | 2 | 2 | 2 | 2 | Sreedharan, et al. 2010 |
| 33 | 2 | 2 | 2 | 2 | 1 | 2 | 1 | 2 | 2 | 2 | 2 | 2 | 2 | 2 | 2 | 2 | 2 | 1 | C. Fuentes, et al. 2010 |
| 31 | 1 | 2 | 1 | 2 | 2 | 2 | 2 | 2 | 1 | 2 | 1 | 2 | 2 | 2 | 2 | 2 | 2 | 1 | Fereshteh Zamani, et al. 2011 |
| 28 | 1 | 2 | 2 | 1 | 2 | 2 | 0 | 1 | 1 | 2 | 2 | 1 | 2 | 2 | 2 | 2 | 2 | 1 | K. Ambak, et al. 2011 |
| 30 | 0 | 2 | 2 | 2 | 2 | 2 | 1 | 1 | 0 | 2 | 2 | 2 | 2 | 2 | 2 | 2 | 2 | 2 | Xuequn, et al. 2011 |
| 26 |  | 2 | 1 | 2 | 1 | 1 | 2 | 1 | 0 | 1 | 2 | 2 | 2 | 2 | 2 | 2 | 2 | 1 | Carlos V R, et al. 2011 |
| 30 | 2 | 1 | 2 | 1 | 2 | 2 | 1 | 2 | 2 | 2 | 1 | 1 | 2 | 2 | 2 | 2 | 2 | 1 | Penprapa Siviroj, et al 2012 |
| 29 | 1 | 2 | 2 | 2 | 1 | 1 | 0 | 1 | 2 | 2 | 2 | 1 | 2 | 2 | 2 | 2 | 2 | 2 | Vatanavongs, et al. 2013 |
| 30 | 1 | 2 | 2 | 2 | 1 | 1 | 1 | 2 | 2 | 1 | 2 | 2 | 2 | 2 | 2 | 2 | 2 | 1 | Jiwattanakulpaisarn, et al. 2013 |
| 31 | 0 | 2 | 2 | 2 | 1 | 1 | 2 | 2 | 2 | 2 | 2 | 2 | 2 | 2 | 2 | 2 | 2 | 1 | Akaateba, et al. 2014 |
| 30 | 0 | 1 | 1 | 1 | 2 | 2 | 2 | 2 | 1 | 2 | 2 | 2 | 2 | 2 | 2 | 2 | 2 | 2 | Casey K. Tsui, et al. 2014 |
| 31 | 2 | 2 | 2 | 2 | 1 | 1 | 2 | 2 | 1 | 2 | 2 | 1 | 2 | 2 | 2 | 2 | 2 | 1 | Moradi G, et al. 2014 |
| 27 | 2 | 2 | 1 | 1 | 2 | 2 | 1 | 1 | 1 | 1 | 1 | 1 | 2 | 2 | 2 | 2 | 2 | 1 | Roksana, et al. 2014 |
| 34 | 2 | 2 | 2 | 2 | 2 | 2 | 1 | 2 | 1 | 2 | 2 | 2 | 2 | 2 | 2 | 2 | 2 | 2 | Rezazadeh J, et al. 2015 |
| 29 | 2 | 2 | 2 | 1 | 2 | 2 | 2 | 1 | 0 | 2 | 1 | 1 | 2 | 2 | 2 | 2 | 2 | 1 | Krishnamurthy, et al. 2015 |
| 29 | 1 | 1 | 2 | 2 | 1 | 1 | 2 | 1 | 0 | 2 | 2 | 2 | 2 | 2 | 2 | 2 | 2 | 2 | Mahdi Quchaniyan, et al. 2015 |
| 28 | 1 | 2 | 2 | 1 | 1 | 2 | 2 | 1 | 0 | 2 | 2 | 1 | 2 | 2 | 2 | 2 | 2 | 1 | Taghi Heydari, et al. 2016 |
| 27 | 0 | 1 | 1 | 2 | 1 | 1 | 2 | 2 | 1 | 1 | 2 | 2 | 2 | 2 | 2 | 2 | 2 | 1 | Grimm M, et al. 2016 |
| 27 | 0 | 1 | 1 | 2 | 1 | 1 | 2 | 2 | 2 | 1 | 2 | 1 | 2 | 2 | 2 | 2 | 2 | 1 | Wadhwaniya S, et al. 2017 |
| 30 | 1 | 1 | 2 | 1 | 2 | 2 | 2 | 2 | 1 | 2 | 1 | 2 | 2 | 2 | 2 | 2 | 2 | 1 | Bachani A, 2017 |
| 30 | 0 | 2 | 1 | 2 | 2 | 1 | 1 | 2 | 2 | 2 | 2 | 2 | 2 | 2 | 2 | 2 | 2 | 1 | Frederick Dapilah, et al. 2017 |
| 30 | 1 | 2 | 1 | 2 | 2 | 2 | 2 | 1 | 0 | 2 | 2 | 1 | 2 | 2 | 2 | 2 | 2 | 2 | Kumphong J, et al. 2018 |
| 30 | 0 | 2 | 2 | 1 | 2 | 2 | 2 | 2 | 2 | 1 | 2 | 1 | 2 | 2 | 2 | 2 | 2 | 1 | Kaviyarasu, et al. 2018 |
| 29 | 1 | 2 | 1 | 2 | 2 | 2 | 2 | 1 | 0 | 2 | 2 | 1 | 2 | 2 | 2 | 2 | 2 | 1 | Jennifer Oxley, et al. 2018 |
| 30 | 0 | 2 | 2 | 1 | 2 | 2 | 2 | 2 | 2 | 1 | 2 | 1 | 2 | 2 | 2 | 2 | 2 | 1 | Eric Nimako Aidoo, et al. 2018 |
| 31 | 1 | 2 | 2 | 2 | 2 | 2 | 2 | 1 | 1 | 2 | 2 | 1 | 2 | 2 | 2 | 2 | 2 | 1 | Ari K.M., et al. 2018 |
| 31 | 0 | 2 | 2 | 1 | 2 | 2 | 2 | 2 | 2 | 1 | 2 | 1 | 2 | 2 | 2 | 2 | 2 | 2 | Adnan, et al. 2019 |
| 33 | 2 | 2 | 2 | 1 | 2 | 2 | 2 | 2 | 2 | 1 | 2 | 1 | 2 | 2 | 2 | 2 | 2 | 2 | Qingfeng Li, et al. 2020 |
| 30 | 0 | 2 | 2 | 1 | 2 | 2 | 2 | 2 | 2 | 1 | 2 | 1 | 2 | 2 | 2 | 2 | 2 | 1 | Jafarian, et al. 2021 |
| 34 | 2 | 2 | 2 | 1 | 2 | 2 | 2 | 2 | 2 | 2 | 2 | 1 | 2 | 2 | 2 | 2 | 2 | 2 | Benjamin, et al. 2023 |
| 34 | 2 | 2 | 2 | 1 | 2 | 2 | 2 | 2 | 2 | 1 | 2 | 2 | 2 | 2 | 2 | 2 | 2 | 2 | Siebert, et al. 2024 |
| 29.98 | 1.05 | 1.77 | 1.74 | 1.51 | 1.63 | 1.63 | 1.6 | 1.65 | 1.19 | 1.63 | 1.84 | 1.44 | 2 | 2 | 2 | 1.98 | 2 | 1.35 | **Mean** |
| *The method for scoring questions according to the STROBE checklist is as follows: - For items that are not mentioned at all in the article text: score 0 - To some extent referred to the item: score 1  - Full mentioned to the suggested items in the text: score 2  **questions:  1- Title and Abstract: A) Using common words to refer to the study type in title or abstract.  B) In the abstract, provide a comprehensive summary of what has been done and achieved.  2. Introduction: Describe the scientific background and aim of the study. 3. The study objectives, including assumptions. 4. Method: Provide key components of the study type in the first sections of the paper.5. Describe the setting, location and dates related to the sampling, fallow up, exposure and data collection. 6. Cross-sectional study: indicate the inclusion criteria, sources and selection methods of participants. 7. Define all the results, exposures, predictor factors, potential confounders, and interactions in a clear manner, and also provide diagnostic criteria.  8. For each variable, indicate the sources of data collection and assessment methods (measurements). If there is more than one group, explain the similarity of the assessment methods. 9- Explain how sample sizes were obtained. 10. Describe all statistical methods, including methods for eliminate the confounding effects.  11. Results: Report the individual’s number at each stage of the study. For example, the number of people who were potentially eligible for study, their conditions confirmed, entered the study, completed the follow-up period, and analyzed. 12. The participants characteristics (such as demographic, clinical and social characteristics) and explain the potential confounding exposures. 13. Cross-sectional study: Report the number of outcome events or summaries.  14. Discussion: mention the key and main findings and objectives of the study. 15. Indicate the study limitations with consideration of possible sources of bias or reduction of accuracy. Discuss about the amount of potential bias. 16- Considering the goals, limitations, statistical analyzes, the results of similar studies and other relevant evidences, provide a comprehensive and prudent interpretation of the results.  17. Discuss the generalizability of the study results (external narrative). 18. Provide funding source and role of financial suppliers in the present study. | | | | | | | | | | | | | | | | | | | |
